# Long-Term Experiences of Health Care Providers Using Iris Scanning as an Identification Tool in a Vaccine Trial in the Democratic Republic of the Congo: Qualitative Study

**DOI:** 10.2196/54921

**Published:** 2025-03-06

**Authors:** Trésor Zola Matuvanga, Antea Paviotti, Freddy Bikioli Bolombo, Gwen Lemey, Ynke Larivière, Maha Salloum, Bernard Isekah Osang'ir, Emmanuel Esanga Longomo, Solange Milolo, Junior Matangila, Vivi Maketa, Patrick Mitashi, Pierre Van Damme, Hypolite Muhindo-Mavoko, Jean-Pierre Van geertruyden

**Affiliations:** 1 Department of Tropical Medicine Faculty of Medicine University of Kinshasa Kinshasa the Democratic Republic of the Congo; 2 Centre for the Evaluation of Vaccination Vaccine and Infectious Disease Institute University of Antwerp Wilrijk Belgium; 3 Global Health Institute Department of Family Medicine and Population Health University of Antwerp Wilrijk Belgium; 4 Division Provinciale de la Santé Ministry of Health Tshuapa Province the Democratic Republic of the Congo

**Keywords:** iris scan, vaccine trial, iris, perception, experience, views, biometric identification, Democratic Republic of the Congo

## Abstract

**Background:**

Iris scanning has increasingly been used for biometric identification over the past decade, with continuous advancements and expanding applications. To better understand the acceptability of this technology, we report the long-term experiences of health care providers and frontline worker participants with iris scanning as an identification tool in the EBL2007 Ebola vaccine trial conducted in the Democratic Republic of the Congo.

**Objective:**

This study aims to document the long-term experiences of using iris scanning for identity verification throughout the vaccine trial.

**Methods:**

Two years after the start of the EBL2007 vaccine trial (February to March 2022), 69 trial participants—including nurses, first aid workers, midwives, and community health workers—were interviewed through focus group discussions. Additionally, 13 in-depth individual interviews were conducted with physicians involved in the trial, iris scan operators, trial staff physicians, and trial participants who declined iris scanning. Qualitative content analysis was used to identify key themes.

**Results:**

Initially, interviewees widely accepted the iris scan and viewed it as a distinctive tool for identifying participants in the EBL2007 vaccine trial. However, over time, perceptions became less favorable. Some participants expressed concerns that their vision had diminished shortly after using the tool and continued to decline until the end of the study. Others reported experiencing perceived vision loss long after the trial had concluded. However, no vision impairment was reported as an adverse event or assessed in the trial as being linked to the iris scan, which uses a previously certified safe infrared light for scanning.

**Conclusions:**

Our findings highlight the sustained acceptability and perceived high accuracy of the iris scan tool for uniquely identifying adult participants in a vaccine trial over time. Continued efforts to systematically disseminate and reinforce information about the function and safety of this technology are essential. Clearly presenting iris scanning as a safe procedure could help dispel misconceptions, concerns, and perceived risks among potential users in vaccine trials.

## Introduction

In many low- and middle-income countries (LMICs), the lack of reliable patient identification can hinder the effectiveness of routine medical care [1-3]. In rural settings, the digitization of personal information and its integration into databases remain limited. Additionally, challenges in identifying patients enrolled over long periods (eg, in longitudinal cohort studies) can lead to misclassification and pose a significant barrier to maintaining study data integrity. A recurring issue during (un)scheduled study visits is that participants often lack a consistent form of identification—whether due to misplacement, forgetfulness, or deterioration. Recently, biometric recognition has gained importance as a tool for accurately identifying individuals in routine health information processes [4-6]. In clinical trials, the use of human biometric recognition has also expanded over the past decade, with adoption becoming increasingly widespread due to its advantages over traditional identification methods [4,7].

Biometric recognition involves a process that relies on the technical processing of data related to the physical, physiological, or behavioral characteristics of the human body (including movement) for authentication purposes [8]. Among the biometric recognition methods used to date—such as fingerprints, facial recognition, iris scans, ear biometrics, and voice recognition—fingerprints are the most widely utilized and have become the predominant form of biometric data [9]. However, unlike fingerprints, which lose clarity over time due to the widening of ridge gaps, the distinct patterns within the iris remain unchanged throughout a person’s lifetime, making iris scan identification exceptionally stable [10-12]. Even among identical twins or between an individual’s own eyes, iris patterns are unique, ensuring their suitability as a reliable proxy for identification [13]. Additionally, iris scanning offers the advantage of being a noncontact process, eliminating the risk of disease transmission through direct contact. As a result, it has the potential to become an affordable, fast, and reliable identification tool across various contexts, including electoral voting, access control, and vaccine trials [4,5,14,15]. In clinical trials, the use of iris scans could help prevent errors and fraudulent entries while ensuring unique participant identification across (un)scheduled visits, thereby safeguarding the integrity of trial results [5]. However, experience with iris scanning as a method of identity verification in clinical trials remains limited.

Recently, the implementation of the iris scan tool demonstrated high precision and strong acceptance among health care providers and frontline worker participants (Multimedia Appendix 1), who were interviewed both before and at enrollment in an Ebola vaccine trial (hereafter referred to as the “EBL2007 vaccine trial”) conducted in a remote area of the Democratic Republic of the Congo (DRC) [4]. This new qualitative research aimed to document long-term experiences with iris scanning for identity verification throughout the trial.

## Methods

### Study Design

This study used a qualitative methodology, specifically a phenomenological approach, to explore experiences related to the long-term use of iris scanning as an identification tool during (un)scheduled visits throughout the EBL2007 vaccine trial.

### Research Team and Reflexivity

The research team comprised TZM and FBB, both male medical doctors and PhD candidates at the University of Kinshasa (DRC), under the coordination of AP, a female social scientist (PhD) from the University of Antwerp (Belgium). TZM and AP developed the interview and topic guides. TZM and FBB conducted the interviews and focus group discussions (FGDs), also facilitating translation between French and Lingala and taking notes as needed. EEL, a male MD from the Provincial Health Division in Boende (Ministry of Health), provided logistical support and assisted with recruitment. Previously, TZM and EEL also served as subinvestigators and trial site coordinators for the EBL2007 vaccine trial.

### Study Setting and Time

This qualitative study on iris scan perception was nested within the EBL2007 vaccine trial (the main study) and conducted in the Health District of Boende, located 1200 km from Kinshasa, the capital of the DRC. Conversations took place on the premises of the General Referral Hospital of Boende (Hôpital Général de Référence de Boende, HGR), which served as the main trial site.

**Figure 1:**
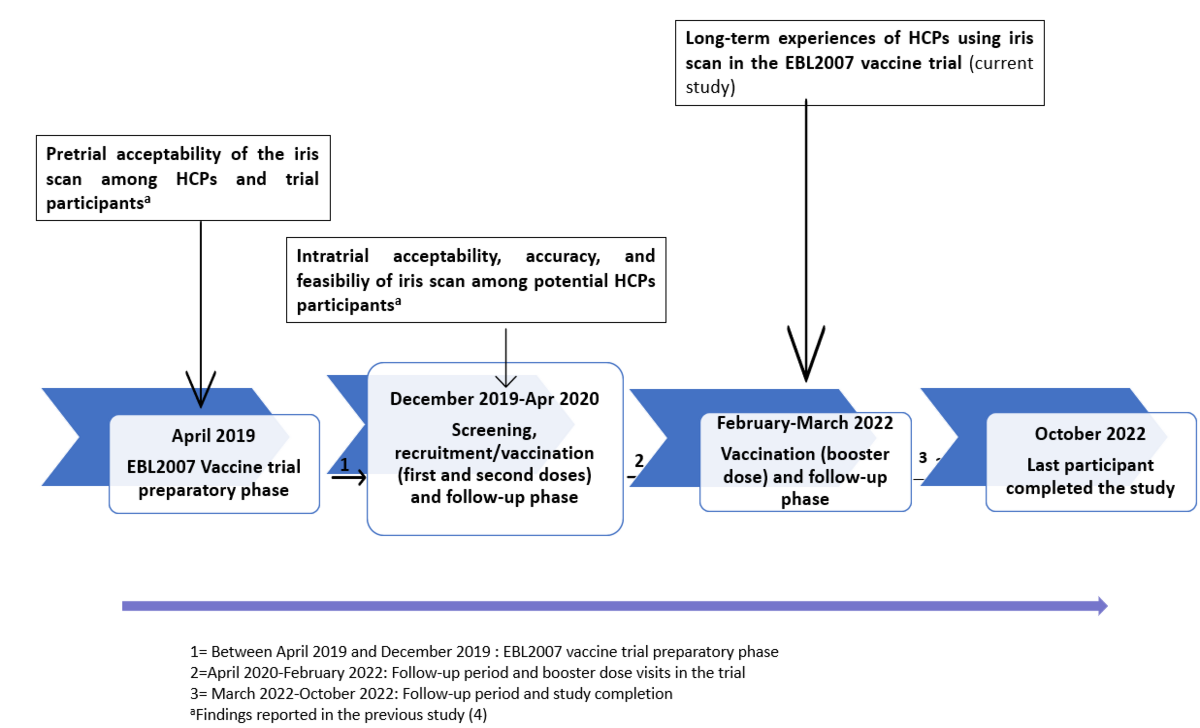
Chronology of events in the EBL2007 vaccine trial. HCPs: health care providers.

Participant recruitment for the main study began in December 2019, with the final patient visit occurring in October 2022 (Figure 1). During the screening process, potential vaccine trial participants were invited to opt into an innovative identification method—an iris scan that captures biometric data from the iris for personal identification. They were informed that this tool was safe and would enable more accurate recognition at both scheduled and unscheduled trial visits.

This qualitative study was conducted between February and March 2022, during the second follow-up period of the main study, when most participants had already completed their last scheduled visit.

Long-term experiences refer to users’ perceptions of the operational viability of the iris scan after 2 years of systematic use within the EBL2007 vaccine trial. Further details on the vaccine trial are reported elsewhere [16].

### Participants’ Recruitment and Sampling

In this qualitative study, purposive sampling was used to include a diverse group of participants and staff from the EBL2007 vaccine trial as it neared completion. The study included the following groups: participants who used iris scanning for identification, participants who initially opted out of iris scanning (including various health care providers involved in the main study, such as nurses, doctors, midwives, first aid workers, community health workers, and health facility cleaners), physician staff involved in the trial, and iris scan operators. Potential study participants were purposively selected through referrals based on their professional category, their consent or refusal to use the iris scanning system, and their level of involvement in its implementation during the main study.

### Data Collection Methods

A combination of FGDs and in-depth individual interviews (IDIs) was applied to collect data from these participants.

Trial participants who had used iris scanning as their identification method from the outset were invited to participate in FGDs. These participants represented a diverse range of professional categories, including community health care workers, who serve as intermediaries between health services and the local population by disseminating information, providing health education, identifying early disease cases, promoting positive health practices, and encouraging individuals to seek professional health care. First aid workers, trained to provide immediate care before professional medical services become available, were also included, along with midwives, nurses, and health facility cleaners. To facilitate open dialogue within each FGD, considerations were given to both the sex and professional categories of the participants.

Furthermore, IDIs were conducted to gather insights from trial participants who had initially opted against using iris scanning for identification. Physicians involved as trial participants and iris scanning operators were also invited to participate in these IDIs.

The selection of trial participants who declined iris scanning was based on documented refusals, as established in prior research [4]. The primary reason for refusing iris scanning among these participants was concern about ocular safety.

### Procedure

For both IDIs and FGDs, a semistructured questionnaire was used (Multimedia Appendix 2). FGDs were conducted in groups of 6-10 participants. Before commencing the IDIs or FGDs, interviewees were reminded of the study’s purpose. Each interview lasted between 60 and 90 minutes, and the conversations were recorded after obtaining the interviewees’ consent.

Voice recordings in languages other than French were translated into French and then into English before being transcribed by 2 independent individuals. The accuracy and coherence of the transcriptions were thoroughly verified by AP and TZM. TZM coded all transcripts derived from both FGDs and IDIs, and AP and TZM reached a consensus on the coding system and categories used for thematic analysis.

### Analysis

This study follows the Consolidated Criteria for Reporting Qualitative Research (COREQ) guidelines to ensure rigor and transparency in the design, data collection, analysis, and reporting of findings (Multimedia Appendix 3). The audio recordings of all conversations were transcribed, translated into French if necessary, and imported into NVivo software (QSR International) for analysis. Each transcript was coded and assigned a unique identifier to ensure confidentiality. AP and TZM held regular collaborative meetings to ensure consistency in the coding process.

The data underwent a thematic analysis approach, beginning with an initial phase of familiarization with the data set. The coding process combined both inductive and deductive approaches [17,18]. Initially, a deductive approach was applied, with codes derived directly from the 2 main themes predetermined in the interview guide: (1) the acceptability of the iris scan and (2) knowledge, perception, and use of the iris scan. The initial codes addressing similar themes were grouped into these predetermined themes using a start list [19]. As the analysis progressed, an inductive approach was incorporated, allowing for the identification of new, unanticipated codes. The start list was subsequently updated to integrate these emergent codes. An iterative process was used to refine and develop subthemes and themes, ensuring alignment with the study objectives. The final themes were substantiated by significant quotations from the transcripts. The analysis was conducted in French to preserve the authenticity and nuance of participants’ responses.

### Trustworthiness

To ensure trustworthiness in our qualitative analysis, we implemented multiple strategies aligned with the criteria outlined by Creswell and Poth [20], as well as the principles proposed by Braun and Clarke [21]. Specifically, credibility was strengthened through source triangulation by integrating data from IDIs, FGDs, and observations across different professional and gender roles within the trial. This methodological approach facilitated the comparison of diverse perspectives, ensuring a more comprehensive understanding of participants’ experiences.

Reflexivity was explicitly addressed in a dedicated section that examined the roles of the investigators throughout the research process. Additionally, verbatim quotes from participants were incorporated into the text to preserve the authenticity of their perspectives and enhance the depth of the analysis.

To enhance transferability, we explicitly detailed our working process and methodology, enabling other researchers to understand and potentially replicate the conditions and results of our study.

Confirmability was strengthened by using multiple data collection methods and engaging independent transcribers and translators to ensure neutrality. Each transcript was meticulously reviewed by the researchers to confirm the accuracy and minimize researcher bias.

Finally, dependability was ensured by maintaining a consistent methodological approach throughout the study, with exhaustive documentation to allow verification of the research process.

We believe that the aforementioned measures provide a solid framework to ensure the reproducibility of our findings.

### Iris Scan Equipment and Procedures

The operator responsible for the iris scanning procedure was a trained and authorized EBL2007 vaccine trial staff member. The iris scan was conducted using an iris camera (Iritech; Irishield Monocular) in conjunction with a tablet (Samsung Tab Active 2) connected to a local ruggedized server via Wi-Fi. Further details regarding the procedures are described elsewhere (Multimedia Appendix 4) [4].

The iris scanner used in the EBL2007 vaccine trial was certified as safe for use with infrared light under all operating conditions, in accordance with the international standard IEC (International Electrotechnical Commission) 62471:2006-07 [22]. Additionally, its irradiance was less than 2% of the Eye Safety Standard Regulation and was tested for photobiological safety.

At enrollment, a list of trial participants who consented to the use of the iris scanning tool for identification during trial visits was compiled. Based on this list, the iris scan operator was instructed not to perform iris scans on participants who had not provided consent. For these individuals, an alternative identification method was used. Their unique participant identification number, assigned at trial enrollment, was recorded, and only their demographic data and identification photo were captured using the iris scanning tool’s tablet. During subsequent (un)scheduled visits, entering their identification number into the tablet allowed for participant recognition based on the stored photograph. For consenting individuals, the operator collected demographic data, took an identification photo, and scanned both irises (left and right eye) as the primary method of identification.

### Ethics Approval

This research was conducted in accordance with ethical principles designed to protect the rights and welfare of all participants. Approval to conduct the study was granted by the National Ethics Committee of Health of the DRC (approval reference number 368/CNES/BN/PMMF/2022). Explicit oral informed consent was obtained before any data collection, following a clearly defined protocol. Interviewees were fully informed about the study’s objectives, the use of their personal data, potential risks and benefits, and their rights to confidentiality and voluntary participation. Consent was recorded, and discussions proceeded only after confirmation. This study had its own protocol and was not a secondary analysis of existing data; therefore, the informed consent process was specific to this research. To protect participants’ privacy and confidentiality, all data were anonymized during transcription and analysis. Unique identifiers were used in place of personal information, and no identifying details were included in the reported findings. Interviewees did not receive monetary compensation for their participation. However, refreshments and transportation allowances were provided to facilitate their involvement in FGDs and IDIs. This was disclosed to participants before they provided consent and was approved by the National Ethics Committee.

## Results

### Participant Recruitment and Data Saturation

Interviews were concluded once data saturation was achieved, with a total of 82 trial participants and staff included in the study. Data saturation was determined when no new themes emerged from subsequent discussions. A total of 69 trial participants took part in FGDs, representing community health care workers, first aid workers, midwives, nurses, and health facility cleaners (Table 1). Additionally, IDIs were conducted with 6 trial participants who refused iris scanning, 5 medical doctors responsible for safety monitoring in the trial, and 2 iris scanning operators.

**Table 1:** Data collection activities for the long-term experiences of health care providers using iris scanning in the EBL2007 vaccine trial.

Interviewees and data collection method		Number of activities, n	Interviewees occupation	Male (n=42), n	Female (n=40), n	Total (N=82), n
**Trial participants**						
	Focus group discussion	2	Nurses	10	8	18
	Focus group discussion	2	First aid workers	9	8	17
	Focus group discussion	2	Community health workers	8	7	15
	Focus group discussion	1	Midwife	0	9	9
	Focus group discussion	1	Health facility cleaner	2	8	10
	In-depth individual interview	1	Physician	3	0	3
	In-depth individual interview	6	Entered the trial but refused iris scan (participants A, B, C, D, E, and F)^a^	6	0	6
**Staff in the trial**						
	In-depth individual interview	2	Physician monitoring safety worker	2	0	2
	In-depth individual interview	2	Iris scan operator	2	0	2

^a^Participants referred to as A, B, C, D, E, and F represent those who chose not to use the iris scanner for identification in the EBL2007 vaccine trial.

Participants had an average age of 51 (SD 11) years. At least one representative from each main professional category enrolled in the EBL2007 vaccine trial participated in either the IDIs or FGDs. The study included 40 (49%) female EBL2007 vaccine trial participants and staff (N=82; Table 1). Three key themes emerged from the collected data: (1) long-term experiences of using iris scanning as an identification tool in the EBL2007 vaccine trial, (2) the potential use of iris scanning in future vaccine trials, and (3) comparisons between iris scanning and previously known identification tools. Within each theme, additional subthemes and categories were identified.

### Long-Term Experiences of Health Care Providers Using Iris Scan as an Identification Tool in the EBL2007 Vaccine Trial

#### Purpose-of-Use Understanding of Trial Participants Who Opted to Use Iris Scan

Some respondents demonstrated a clear understanding of the rationale behind using iris scanning, as reflected in the following statements:

It helped us because it brought out the whole face.

FGD, midwife, trial participant, woman

It was for identification purposes.

FGD, midwife, trial participant, woman

However, a few respondents had a different understanding of the iris scanning tool, perceiving it as a method for vaccine trial investigators to detect disease or assess the impact of the experimental vaccine on the eyes.

Me, I believed they’re going to find the disease in the eyes, and they’re going to tell us, but they haven’t told us, and we haven’t asked.

FGD, midwife, trial participant, woman

#### Acceptability of the Iris Scan

In general, health care providers and frontline workers volunteering in the EBL2007 vaccine trial widely accepted the use of iris scan technology, consistent with findings from the start of the trial [4]. One community health worker mentioned:

[...] I haven’t come across anyone who would tell me that they didn’t accept it. You see, I haven’t encountered any group of people or any individual who would refuse to be examined by this device.

FGD, community health worker, trial participant, man

#### Reasons for Accepting the Iris Scan

The primary reason most interviewees accepted the iris scan was their willingness to receive the study vaccine. Their confidence in the trial staff further motivated them to trust the procedures proposed in the trial.

We accepted it because we were looking at our study vaccine, we were looking at the advantage of being enrolled in the Ebola vaccine trial.

FGD, nurse, trial participant, man

However, some respondents stated that they adopted the iris scan because they understood it as the appropriate identification tool for accurately recognizing participants enrolled in the trial.

We have observed that it is a valuable tool for identification, which is why it was accepted.

FGD, first aid worker, trial participant, woman

#### Reasons for Not Accepting the Iris Scan

A handful of interviewees indicated that they did not feel comfortable with the tool due to concerns about the retention of their demographic data recorded by it.

Yes, for a psychological reason, for example, we might take this photo and put it in a book, in a documentary, so that people can see you. So that’s why I refused. But as I didn’t have any information, I couldn’t accept.

FGD, community health worker, trial participant, man

Some interviewees, due to the condition of their eyes before the use of the iris scan, expressed a fear of vision loss associated with the tool, compounded by their apprehension upon seeing it for the first time.

I refused because I’m sick. My eye hurts, especially my left eye, which has been bothering me since 2012. I’ve been suffering for four years. It’s in this sense that I refused because the way it was flashing, it may burn my eye. Especially when she filmed it, it scared me. That’s why I refused.

Interview, trial participant B who refused iris scan

#### Purpose-of-Use Understanding of Trial Participants Who Declined the Use of Iris Scan

Some interviewees who declined iris scan identification reported that they were unaware of the rationale for using this tool in the vaccine trial.

Well, I don’t know the importance of this.

Interview, trial participant A who refused iris scan

I still have doubts, but I’ve seen the people who have had iris scans, and I don’t see the point in continuing to doubt, given that there are friends who have accepted, and they’re still here.

Interview, trial participant E who refused iris scan

In my opinion, regarding the importance of iris scan, when they captured us, I explained that my eye is diseased, but they told me they would capture the other eye, and I refused. But I don’t know the significance of it.

Interview, trial participant F who refused iris scan

Some interviewees reported understanding that the scanning tool was intended for identification purposes during visits to prevent possible fraud.

[...] We mentioned that we would use it to confirm the person’s identity. If someone else tries to pretend to be me, when they put the iris scan in their eye, it will show that it is not them. That is what I remember about the iris scan.

Interview, trial participant E who refused iris scan

#### The Perceived Accuracy of Iris Scan in Identifying Participants

Most interviewees reported that they found the iris scan tool in the trial to be highly accurate, as it helped prevent cases of fraud among participants. They provided examples of fraud attempts to illustrate the tool’s effectiveness and accuracy. One such example is the following statement:

[...] There was an incident where I arrived for a scheduled visit and the person sitting close to me presented his father’s identification card, claiming to be him. Upon verification, the operator discovered that the person in front of him was not who he claimed to be. The photo in the system of iris scan did not match his appearance. He eventually admitted that his father had passed away. The individual conducting the check informed him that we had not been informed of the father’s passing, and upon further investigation, it was revealed that the father was traveling and had authorized his son to represent him. This incident revealed potentially fraudulent activity, as the person attempted to manipulate the results of the vaccine trial by assuming someone else’s identity.

FGD, community health care worker, trial participant, woman

#### Perceived Risk of Iris Scan Identification

Some participants perceived a link between the use of the iris scan and vision loss. Additionally, most participants expressed concerns that vision loss might occur later due to the amount of light emitted by the device.

Certain interviewees compared the light emitted by the iris scan to the eclipse phenomenon or sunlight, suggesting that this may have contributed to vision loss.

We were scared, there was the light and there was uncertainty as to whether the eye would crack or not [...]

FGD, community health care worker, trial participant, man

Thank you, now after the iris scan, I have noticed that there is a reduction of vision, especially for reading, so we have to use glasses now.

FGD, first aid worker, trial participant, man

Certain interviewees mentioned that they did not observe any unusual occurrences during the scanning process and expressed no apprehension about the future safety of their eyes, as they did not perceive the iris scan as hazardous.

There was no reaction. They just tell you to stare like this and then they tell you, it’s okay. There wasn’t really any direct reaction like that.

FGD, nurse, trial participant, woman

I was just afraid for my eyes, but it is not dangerous. Even if it comes back to my village, we will make people aware of this device. It is for identifying people.

Interview, trial participant E who refused iris scan

#### Rumors and Reactions From the Surroundings

Certain interviewees and FDG expressed concerns about the well-being of their eyes based on conversations. Nevertheless, most emphasized that they had not heard any rumors related to the iris scan. Instead, the rumors primarily focused on the experimental vaccine and other study procedures, such as blood sampling.

They said your eye is sick if you use this device, your eye will be completely damaged, which is why I was afraid. Otherwise, there wasn’t much to it.

Interview, trial participant B who refused iris scan

No, in the neighbourhoods there hadn’t been any rumours, but it was about the vaccine and blood sampling that people were talking nonsense about, not about the iris scan.

FGD, first aid worker, trial participant, man

The people around us didn’t know that we were having the iris scan in the study, they only knew that we were selling our blood and getting vaccinated, period, but concerning the Iris scan, nothing was said, it was only us, trial participants, who knew about the Iris scan, but not the community, they didn’t know anything about it. In the neighbourhood, we were nicknamed blood sellers.

FGD, health facility cleaner, trial participant, woman

#### Use of Iris Scans in Future Vaccine Trials or Other Public Health Activities

##### Acceptability of Iris Scan in the Wider Community

As the iris scan tool is part of a vaccination monitoring system that collects additional data (eg, demographics, a photo of the face) beyond those recorded for routine vaccinations (eg, demographics, previous vaccinations, the name of the vaccine administered, its lot number, and expiry date), certain participants believed that the iris scan tool would neither be accepted nor feasible in broader vaccination efforts involving the general population. Participants referenced a yellow fever vaccination campaign that had taken place in Boende. During this campaign, people agreed to receive the vaccine, but many were unwilling to provide full identities and demographic data. Therefore, some interviewees and FGD participants suggested that a different tool, such as fingerprint scanning, would be preferable instead.

I wanted to say that for the population, it’s going to be a bit difficult, because we’ve noticed here that with yellow fever, we only recorded the name on the card and then gave the vaccine directly. It was also difficult to get someone to agree to give their full identity so that they could be vaccinated, so it would also be very difficult with the iris scan. It’s better even with the fingerprint, maybe it will be all right. With the iris scan, it will be a bit difficult with this population.

FGD, nurse, trial participant, woman

Some interviewees emphasized that iris scanning could be an acceptable, effective, and reliable tool for uniquely identifying individuals who might volunteer in future clinical trials. However, they stressed that this should be accompanied by a robust awareness campaign to repeatedly communicate sufficient information about the tool’s safety.

At first, people will refuse, but after awareness-raising and testimonials from those who have experienced the tool, they will accept.

Interview, trial participant A who refused iris scan

##### Recommendations From Interviewees and FGD Participants

When implementing iris scanning in vaccine trials, particularly in remote areas such as Boende, some interviewees and FGD participants recommended considering the availability of ophthalmic specialists. According to their statements, the iris scan operator in the EBL2007 vaccine trial was not fully aware of the risks involved in scanning the eyes and was unable to provide clear explanations regarding the safety of trial participants’ eyes.

[...] when you come to scan people’s eyes, come with the eye specialist. [...]. All those who have handled our eyes are not specialists. [...] They are photographers, so you should to come with eye specialists. An ophthalmologist because it’s a sensitive organ.

FGD, first aid worker trial participant, trial participant, man

Few of those who participated in the FGDs voiced concerns about not seeing the vaccine trial investigators undergo iris scanning.

[...] Until now we haven’t seen the staff being vaccinated, or scanned the eyes with iris scan. We haven’t seen; they haven’t scanned themselves.

FGD, first aid worker, trial participant, man

Some participants in this research recommended reducing the amount of light used during scanning, increasing the distance between the eye and the scanner, and conducting demonstrations during the screening/consent process.

I think that, as my colleague the Community health worker just said, the distance from the iris scan is too close. Isn’t there some way of finding ways of making it even bigger? [...].

FGD, community health worker, trial participant, woman

##### Comparison of Iris Scan With Previous Known Identification Tools

Referring to previously used and well-known identification methods, most interviewees stated that iris scanning would be the best way to uniquely identify volunteers in the trial.

With the experience that I have, with the age that I have...I believe that the only method of escaping fraud is scanning [...]

FGD, community health worker, trial participant, woman

[...] So, with today’s technology, we may easily modify the photo by taking someone’s face and putting it on another body to make it look like it’s me, but it’s not. But with the iris scan, it’s easy to see that it’s not me, it’s just someone else’s face. So, with the iris scan, it’s hard to commit fraud.

FGD, community health worker, trial participant, woman

Some interviewees suggested using the traditional fingerprint biometric tool to minimize the risk of compromising their eyes with iris scanning or deploying other methods, such as recording names, dates of birth, and identification numbers in a computer. Others proposed more innovative identification tools, such as collecting identity data through the laser thermometer used to measure temperature at entry points or utilizing blood samples already collected during the first visit.

I’m going to recommend the fingerprint because the signature may be imitated, but your fingerprint, your own blood, will reveal all your data.

FGD, community health worker, trial participant, man

[...] instead of using this device, there was no way of using our fingerprints. Because...Isn’t it possible to use a fingerprint?

FGD, community health worker, trial participant, man

[...] As we’ve received all the doses of vaccine as well as blood samples were taken, it’s the computer that will indicate that for such and such a participant, he’s finished his doses, these appointments are over, so that’s it. The computer is a method.

FGD, health facility cleaner, trial participant, woman

[...] We may have a thermometer that records all the identity as well as the blood pressure and everything, so as not to have any problems with the use of that iris scan laser.

FGD, community health worker, trial participant, man

## Discussion

### Principal Findings

This qualitative study aimed to document the long-term experiences of EBL2007 vaccine trial participants and staff regarding the use of an innovative iris scan biometric tool. Overall, the tool was found to be acceptable, accurate, and effective in verifying participants’ identities throughout the trial, preventing fraud and errors.

Although it was clearly explained during the consent procedure that the iris scan was noncompulsory, some participants may have feared that declining it would prevent their enrollment. Similarly, the safety of the iris scan was thoroughly explained to trial personnel at the start of the trial. Despite these efforts, some interviewees and FGD participants still felt that scanning their eyes posed safety risks or that it might cause problems in the future.

It is important to highlight the expected motivational benefits of participating in the EBL2007 vaccine trial, as some interviewees may have accepted the iris scan primarily to receive the Ebola vaccine regimen, given that they reside in an area at risk of an outbreak [23], or to obtain reimbursement for travel costs and time associated with the trial [24]. Additionally, concerns about eye safety after using this tool should not be overlooked. Various vision problems were perceived to be associated with the iris scan.

In studies conducted elsewhere, similar reasons for hesitancy—such as general safety concerns and anxiety about the physical effects of biometric scanning—have been reported [14,25,26]. However, it is important to note that vision impairment was neither reported as an adverse event nor assessed as being associated with iris scanning during the trial. Additionally, concerns were raised regarding the collection and safeguarding of additional personal data following the iris scan. Notably, collecting such information was new to trial participants in the remote area of Boende (the trial site), where the most common biometric practice—used for payroll verification, passport applications, and voter registration—remains fingerprinting [27]. This familiarity with fingerprinting may have contributed to greater comfort with it compared with iris scanning and may have also raised security concerns about iris scanning, despite its general acceptance and perceived accuracy.

It is likely that the vision disorders perceived to be associated with iris scanning in the EBL2007 vaccine trial had other causes. Some participants may have had preexisting eye conditions. For instance, while the mean age of trial participants was 45 years [27], it is well known that the incidence of vision impairment increases from middle age onward [28,29]. Additionally, at the time of the EBL2007 vaccine trial, no ophthalmological care was available in Boende. As a result, participants’ vision or ophthalmological complaints may not have been addressed at the time of enrollment. Promotional and preventive activities aimed at improving eye health may also be necessary, as studies have shown that visual impairment is prevalent in populations living in remote, resource-constrained areas due to limited access to quality health care services [30]. However, implementing such activities may introduce additional costs for researchers. Given the increasing spread of digital legislation and democracy in Africa [31], these concerns may gradually diminish with the wider use of iris scanning technology. This suggests that even if research participants develop vision problems—likely due to aging or other factors—they may not attribute them to iris scanning as digital technology becomes more commonplace.

Some interviewees noted that the iris scanner operator appeared more like a photographer than someone capable of properly explaining the tool and its safety information. This suggests that the purpose and function of the iris scan tool were not sufficiently explained at inclusion or during follow-up visits in the trial. As a lesson learned, it is crucial to provide more detailed training to the iris scan operator to ensure they can answer specific questions from research participants.

It is also worth emphasizing that the timing of this qualitative study—conducted 2 years after the trial began—may explain why some interviewees and FGD participants gradually forgot the information about the iris scan tool provided during the initial consent process. Previous studies have highlighted that trial volunteers often forget or retain less of the information given at the time of enrollment [32,33]. This suggests that in long-term studies, the contents of the informed consent form should be periodically re-explained to participants.

This study demonstrated that iris scanning in vaccine trials conducted in resource-poor settings has valuable potential and is generally accepted for participant identification. The acceptability of iris scanning aligns with the key properties required of a high-performance biometric tool, such as universality, uniqueness, permanence, collectability, and resistance to circumvention [31,34]. However, during the EBL2007 vaccine trial, the iris scanning process became cumbersome when identification was not possible due to a participant’s failure to follow the operator’s instructions. To facilitate rapid identification through iris scanning, it is essential that individuals remain attentive and adhere strictly to the operator’s guidance. This challenge may be particularly pronounced in vaccine trials involving younger infants (under 1 year old). Although infants are among the populations most in need of vaccines, they are unable to follow detailed instructions—such as looking into a camera—required for iris recognition [15]. Alternative approaches, such as scanning the iris of the accompanying adult (proxy identification number) or using ear-based or palm-based automatic recognition, may be more suitable for infants and other dependent populations [35,36].

### Limitations

This study has some limitations. First, our findings are based on long-term experiences—spanning multiple iris scanning moments over a 2-year study period—of a population of health care providers and frontline workers who likely have a higher level of understanding of health-related phenomena. As a result, these findings may not be generalizable to the broader population. Second, some of the researchers who conducted FGDs and interviews, although not directly involved in the medical aspects of the vaccine trial, may have been perceived by interviewees as representing the trial team. This perception could have introduced desirability bias in the IDIs and FGDs. Finally, some participants working at the General Referral Boende Hospital—also the study site of the trial—may have hesitated to express negative concerns about the iris scan due to the location of the interviews (ie, at the hospital) or because some interviews were conducted by a trial investigator. Nevertheless, the findings of this qualitative research reflect participants’ and staff’s retrospective experiences with iris scanning over time and complement previous qualitative research on its acceptability. To our knowledge, this study, combined with the earlier research on the acceptability of biometric identity verification tools, provides the only comprehensive analysis of both the initial acceptability and the actual experiences of iris scanning within the same trial population [4]. The insights gained can inform the broader implementation of iris scanning in vaccine trials or other long-term longitudinal research.

### Conclusions

The findings of this qualitative research highlight the sustained acceptability and perceived high accuracy of the iris scan tool for uniquely identifying adult participants in a vaccine trial over time. However, when the functionality of the iris scan is not well understood or remembered by users, certain concerns may arise, including perceived risks to long-term vision, the use of retained data, and the tool’s ability to rapidly verify information regardless of age, education level, or health condition. To support broader implementation in vaccine trials or other research, further efforts should be made to provide clear information to users and dispel misconceptions about the fears and perceived risks associated with the iris scan tool.

## Appendix

Multimedia Appendix 1 The profile of health care providers included in our previous study [4], which evaluated the acceptability of iris scanning in the EBL2007 trial before and during the recruitment process.

Multimedia Appendix 2 The semistructured questionnaire used to interview participants.

Multimedia Appendix 3 Reporting follows the COREQ (Consolidated Criteria for Reporting Qualitative Research) guidelines, which best match our study design to ensure completeness and detail.

Multimedia Appendix 4 The process and the iris scanning tool used as an identification method in the EBL2007 trial, with the user experience—including clinical trial participants, staff, and operators—reported in our qualitative study.

Multimedia Appendix 5 It refers to the collection of transcripts (French) from all conversations (interviews and focus group discussions).

## Abbreviations

DRC: The Democratic Republic of the Congo
FGD: focus group discussion
IDIs: in-depth individual interview
IEC: International Electrotechnical Commission
LMIC: low- and middle-income country

## Data Availability

The data sets generated during this study are available and included as multimedia appendices (Multimedia Appendices 1-5).
