# Supplementary material for: Long-Term Experiences of Health Care Providers Using Iris Scanning as an Identification Tool in a Vaccine Trial in the Democratic Republic of the Congo: Qualitative Study
Source: JMIR Form Res. 2025 Mar 6;9:e54921. doi: 10.2196/54921 (PMC11926449; doi:10.2196/54921)
Supplement: Multimedia Appendix 1 [file formative_v9i1e54921_app1.docx]

**Table 1. Focus group discussion with HCPs potential participants in the EBL2007 vaccine trial for the qualitative acceptability assessment prior the trial^*^**

|  | Method | Interviewees occupation | Male | Female | Total (N) |
| --- | --- | --- | --- | --- | --- |
|  | 4 FGD | Nurses | 22 | 4 | 26 |
|  | 1 FGD | First aid workers worker | 6 | 2 | 8 |
|  | 3 FGD | Community health workers | 20 | 4 | 24 |
|  | 1 FGD | Mid-wives | - | 7 | 7 |
|  | 1 FGD | Cleaners | 1 | 6 | 7 |
|  | 1 FGD | Physician | 7 | 1 | 8 |
|  | 1 FGD | Lab technicians | 5 | 1 | 6 |
| Total |  |  | 39 | 41 | 86 |

* Matuvanga, Z., Trésor, et al. J. Med. Internet Res. 23.8 (2021): e28573.
