# Supplementary material for: Long-Term Experiences of Health Care Providers Using Iris Scanning as an Identification Tool in a Vaccine Trial in the Democratic Republic of the Congo: Qualitative Study
Source: JMIR Form Res. 2025 Mar 6;9:e54921. doi: 10.2196/54921 (PMC11926449; doi:10.2196/54921)
Supplement: Multimedia Appendix 2 [file formative_v9i1e54921_app2.docx]

Semi structured questionnaires

**I. Thematic discussion guide for Focus Groups Discussions with Trial-participants in the EBL2007 vaccine trial**

Theme 1: Knowledge and perception of iris scanning technology

1. What is the purpose of iris scanning technology?

- Why was the iris scanning tool used in this study?

2. How does the iris scanner work?

3. How was the iris scanning tool explained to you?

- Who explained it to you and how?

- What information reassured you the most? What information worried you the most?

4. How did you feel during identification using the iris scanner?

5. How did you like the iris scanning tool used by the project (study EBL2007 vaccine trial) to identify you on the day of your various study appointments?

- After trying it out, do you still have any questions or fears about using this tool? Please describe them.

6. After using the iris scanning tool, was it worth using it in this project, in your opinion?

7. How reliable do you think this method of identification is? What do you think are the reasons for this high/low reliability?

8. How do you think volunteers/participants like you in a clinical trial should be identified during visits to ensure that it is the right person who is in the clinical trial in the future?

- Do you know of any other secure identification tools/means used in other clinical trials?

- Which identification tool would you recommend for future clinical trials? Why or why not?

- If you had the choice between iris scan tool, fingerprints, photo ID or other methods of identification at the start of the EBL2007 vaccine trial, which method would you choose?

Theme 2: Acceptability of the iris scan tool

9. Of those who decided not to be identified using the iris scanning tool, what do you think were the reasons for their decision?

10. What reactions did you experience from your family/community members after being identified with the iris scan tool as a clinical trial participant? Why or why not?

11. Have you heard of any participants being stigmatised here in Boende because of their identification with the iris scan tool?

- How are they stigmatised?

- By whom?

- By whom?

Have there been any rumors in your circle about the iris scanner? What is being said about this tool in the context of the current study?

12. Do you think that other healthcare professionals or future participants in other studies, here or elsewhere, will accept the iris scanning tool as a means of identification? Why or why not?

13. In your opinion, what could be some potential considerations for researchers who plan to use the iris scan tool in future vaccine trials?

- Do you think it will be different or similar for studies conducted in another region of the DRC?

**II. In-depth Interview guide for semi-structured EIAs with participants who refused to be identified using the iris scanning tool**

Theme 1: Knowledge and perception of iris scanning technology

1. What is the purpose of iris scanning technology?

- Why was the iris scanning tool used in this study?

2. How does the iris scanner work?

3. How was the iris scanning tool explained to you?

- Who explained it to you and how?

- What information reassured you the most? What information worried you the most?

4. What were your reasons for refusing identification using the iris scanner?

5. After observing the use of the iris scanner, do you still have the same questions or fears about using this tool?

- If not, what made you change your mind?

6. After observing the use of the iris scanning tool, was it worth using it, in your opinion?

7. Do you think that this technology made it possible to detect cases of fraud that would have been missed using other means usually used to identify people (vaccination card, identity document, etc.)? How and thanks to what would this have been possible?

8. How do you think volunteers/participants like you in a clinical trial should be identified during visits to ensure that it is the right person who is in the clinical trial in the future?

- Do you know of any other secure identification tools/means used in other clinical trials?

- Which identification tool would you recommend for future clinical trials? Why or why not?

Topic 2: Acceptability of the iris scanning tool

9. What reactions would you have experienced from your family/community members if you had been identified with the iris scan tool as a clinical trial participant? Why or why not?

10. Have you heard of any participants being stigmatised here in Boende because of their identification with the iris scan tool?

- How are they stigmatised?

- By whom?

- Why or why not?

11. What is being said about this tool in the context of the current study?

12. Do you think that other healthcare providers or future participants in other studies, here or elsewhere, will accept the iris scanning tool as a means of identification? Why or why not?

13. In your opinion, what could be some potential considerations for researchers who plan to use the iris scan tool in future vaccine trials?

- Do you think it will be different or similar for studies conducted in another region of the DRC?

**III. Interview guide for In-depth individual interview with physicians staff in the trial**

Theme 1: Knowledge and perception of iris scanning technology

1. What is the purpose of iris scanning technology?

- Why was the iris scanning tool used in this study?

2. How does the iris scanner work?

3. How was the iris scanning tool explained to you?

- Who explained it to you and how?

- What information reassured you the most? What information worried you the most?

4. How did you assess the iris scanning tool used by the project (EBL2007 vaccine trial) to identify you on the day of your various study appointments? How did you feel during the identification process using the iris scanner?

- What are your thoughts or feelings about using this tool after having tried it out?

5. After using the iris scanner, what do you really think of it personally? Do you think it was worth using?

6. Do you think that this technology has made it possible to detect cases of fraud that would have been missed using other means usually used to identify people (vaccination card, identity document, etc.)? How and why would this have been possible?

7. How do you think volunteers/participants like you in a clinical trial should be identified during visits to ensure that it is the right person who is in the clinical trial in the future?

- Do you know of any other secure identification tools/means used in other clinical trials?

- Which identification tool would you recommend for future clinical trials? Why or why not?

Theme 2: Acceptability of the iris scanning tool

8. Of those who have decided not to be identified using the iris scanner, what do you think are the reasons for their decision?

9. Did you hear any participants complain about the use of the iris scan tool as being the cause of a problem with the health of their eyes or another part of the body?

- If so, what problem did the participants mention? How was this understood by the participant?

10. In the day-to-day management of patients, did you identify any eye health problems that could be explained using the iris scanning tool in a participant in this clinical trial?

- If so, what was the reaction of those concerned? How do you understand this?

11. What reactions did you experience from your family/community members after being identified with the iris scan tool as a clinical trial participant? Why or why not?

12. Have you heard of any participants being stigmatised here in Boende because of their identification with the iris scan tool?

- How are they stigmatised?

- By whom?

- Why or why not?

13. Have there been any rumours in your circle about the iris scanner? What is being said about this tool in the context of the current study?

14. Do you think that other healthcare professionals or future participants in other studies, here or elsewhere, will accept the iris scanning tool as a means of identification? Why or why not?

15. What are your thoughts on the potential impact of using the iris scan tool in organizing future vaccine trials?

- Do you think these challenges and difficulties will be different or similar for studies conducted in another region of the DRC?

**IV. Interview guide for In-depth individual interview with operators who have handled the iris scanning tool in the trial**

Theme 1: Knowledge, perception and use of iris scanning technology

1. What is the purpose of iris scanning technology?

- Why was the iris scanning tool used in this study?

2. How are volunteers in this clinical study identified using the iris scanning tool?

3. How was the operation of the iris scanning tool explained to you?

- Who explained it to you and how?

- What information reassured you the most? What information worried you the most?

4. From the point of view of handling, what do you think of this tool?

- What was easy and what was difficult during handling?

- What would need to be improved to make this tool easier to use?

- What do you think of its effectiveness in identifying study volunteers? Do you think that this technology made it possible to detect cases of fraud that would have been missed using other means usually used to identify people (vaccination card, identity document, etc.)?

- What aspects could be improved to make this tool more effective?

5. Could you describe your experience when using the tool, including any feelings or reactions you had?

6. How did the participants react when their irises were scanned?

- What questions did participants ask you most often?

- What were their fears?

- Were there any differences in reaction between the first and second visits?

- Which reaction impressed you the most?

7. How do you think volunteers/participants like you in a clinical trial should be identified at future visits to ensure that it is the right person who is in the clinical trial?

- Do you know of any other secure identification tools/means used in other clinical trials?

- Which identification tool would you recommend for future clinical trials? Why or why not?

8. After experimenting with the tool, do you still have questions or fears related to its use? Please describe them.

9. After using the iris scanner, what do you really think of it personally? Do you think it was worth using?

Theme 2: Acceptability of the iris scanning tool

10. Of those who decided not to be identified using the iris scanner, what do you think were the reasons for their decision?

11. Have you heard of any participants being stigmatised here in Boende because of their identification with the iris scanning?

- How are they stigmatised?

- By whom?

- Why or why not?

12. What is being said about this tool in the context of the current study?

13. Do you think that other healthcare professionals or future participants in other studies, here or elsewhere, will accept the iris scanning tool as a means of identification? Why or why not?

14. What are your thoughts on the potential impact of using the iris scan tool in organizing future vaccine trials?

- How might the challenges and difficulties of conducting studies vary across different regions of the DRC, in your opinion?
