# Supplementary material for: Long-Term Experiences of Health Care Providers Using Iris Scanning as an Identification Tool in a Vaccine Trial in the Democratic Republic of the Congo: Qualitative Study
Source: JMIR Form Res. 2025 Mar 6;9:e54921. doi: 10.2196/54921 (PMC11926449; doi:10.2196/54921)
Supplement: Multimedia Appendix 4 [file formative_v9i1e54921_app4.pptx]

## Slide 1
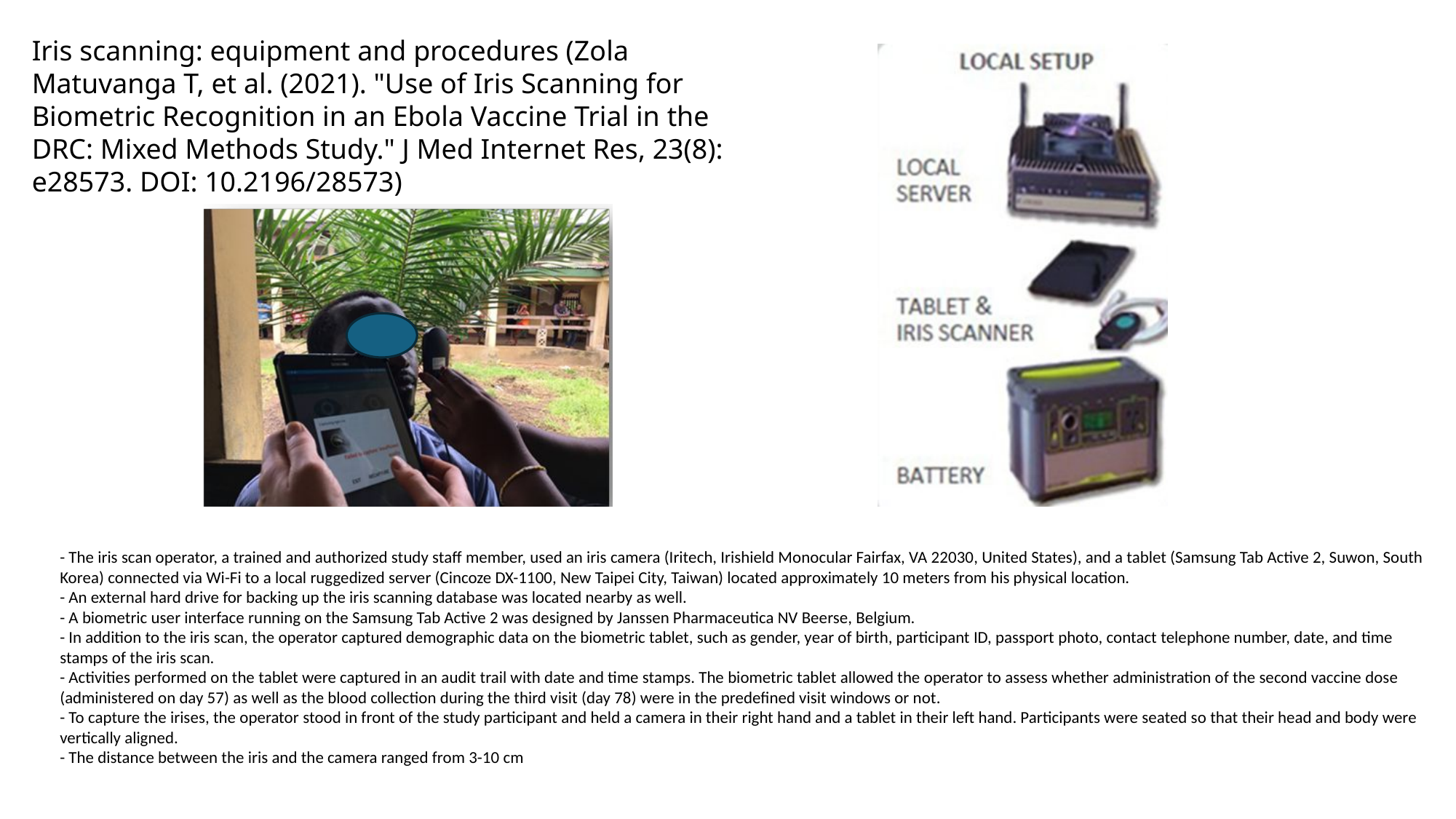

Iris scanning: equipment and procedures (Zola Matuvanga T, et al. (2021). "Use of Iris Scanning for Biometric Recognition in an Ebola Vaccine Trial in the DRC: Mixed Methods Study." J Med Internet Res, 23(8): e28573. DOI: 10.2196/28573)
- The iris scan operator, a trained and authorized study staff member, used an iris camera (Iritech, Irishield Monocular Fairfax, VA 22030, United States), and a tablet (Samsung Tab Active 2, Suwon, South Korea) connected via Wi-Fi to a local ruggedized server (Cincoze DX-1100, New Taipei City, Taiwan) located approximately 10 meters from his physical location.
- An external hard drive for backing up the iris scanning database was located nearby as well.
- A biometric user interface running on the Samsung Tab Active 2 was designed by Janssen Pharmaceutica NV Beerse, Belgium.
- In addition to the iris scan, the operator captured demographic data on the biometric tablet, such as gender, year of birth, participant ID, passport photo, contact telephone number, date, and time stamps of the iris scan.
- Activities performed on the tablet were captured in an audit trail with date and time stamps. The biometric tablet allowed the operator to assess whether administration of the second vaccine dose (administered on day 57) as well as the blood collection during the third visit (day 78) were in the predefined visit windows or not.
- To capture the irises, the operator stood in front of the study participant and held a camera in their right hand and a tablet in their left hand. Participants were seated so that their head and body were vertically aligned.
- The distance between the iris and the camera ranged from 3-10 cm
