# Supplementary material for: Long-Term Experiences of Health Care Providers Using Iris Scanning as an Identification Tool in a Vaccine Trial in the Democratic Republic of the Congo: Qualitative Study
Source: JMIR Form Res. 2025 Mar 6;9:e54921. doi: 10.2196/54921 (PMC11926449; doi:10.2196/54921)
Supplement: Supplementary file 5 [file formative_v9i1e54921_app5.docx]

5.1.1.2- EIA staff ayant manipul iris scan 1- pa	5.1.1.2.FGD- RECO- H- OK.docx	5.1.1.2.FGD- RECO- H. docx	5.1.1.2.FGD- RECO- F.d ocx	5.1.1.2.FGD- Infirmiers -H.docx
5.1.1.2.FGD- Infirmière s-F.docx	5.1.1.2.FGD.Infirmiers Hommes.docx	5.1.1.2.FDG- Sécourist es-Femmes.docx	5.1.1.2.FDG- Netoyeur s-mixte.docx	5.1.1.2.EIA- Staff- Med ecin-suivi de la sécurit
5.1.1.2.EIA- Participant ayant refusé iris scan-	5.1.1.2.EIA- Médecin-p articipant EBL2007-2.c	5.1.1.2.EIA- Médecin-p articipant EBL2007-1.c	5.1.1.2.EIA.Staff opérateur iris-1-parti	5.1.1.2.EIA.Participant ayant refusé iris scan-
5.1.1.2.EIA.Participant ayant refusé iris scan-	5.1.1.2.EIA.Participant ayant refusé iris scan-	5.1.1.2.EIA.Participant ayant refusé iris scan-	5.1.1.2. FDG- Matronnes (Sage	5.1.1.2 FGD- Sécouristes- H.do
5.1.1. EIA Médecin - participant -EBL2007.c	5.1.1.2- EIA.Staff opérateur iris-2-.docx			
